# The association between fruit and vegetable consumption and metabolic syndrome in Korean adults: does multivitamin use matter?

**DOI:** 10.4178/epih.e2022039

**Published:** 2022-04-19

**Authors:** Jihae Kim, Li-Juan Tan, Hyein Jung, Yumi Roh, Kyungjoon Lim, Sangah Shin

**Affiliations:** 1 Department of Food and Nutrition, Chung-Ang University, Anseong, Korea; 2 Faculty of medicine and Health, School of Medical Science, University of Sydney, Sydney, Australia

**Keywords:** Dietary behavior, Metabolic syndrome, Vitamins, Adults, Cohort studies

## Abstract

**OBJECTIVES:**

Metabolic syndrome (MetS) is closely associated with dietary intake; however, few studies have investigated whether the consumption of fruits and vegetables and multivitamin use affect MetS in the Korean population. This study aimed to examine these effects in Korean adults.

**METHODS:**

This was a cross-sectional study of 89,548 participants aged between 40 years and 69 years selected from the baseline data of the Health Examinees study conducted in Korea. Fresh vegetable and fruit consumption was assessed using a validated 106-item food frequency questionnaire. MetS and its components were defined using the National Cholesterol Education Program, Adult Treatment Panel III criteria. Multivariate logistic regression analyses were conducted to identify associations of fresh vegetable, fruit, and fresh vegetable+fruit consumption and multivitamin use with the prevalence of MetS.

**RESULTS:**

Female in the highest quartile of fresh vegetable, fruit, and fresh vegetable + fruit consumption exhibited a lower prevalence of MetS than those in the lowest quartile. An inverse association with the prevalence of MetS was observed among male with only fresh vegetable consumption. The interaction between the 3 categories and multivitamin intake on the prevalence of MetS was not significant (all p_interaction_>0.05), regardless of sex.

**CONCLUSIONS:**

Multivitamin use and consumption of fresh vegetables and fruits had no significant synergistic effects. Although fresh vegetable and fruit consumption showed an inverse association with the prevalence of MetS, this relationship was not altered by multivitamin use.

## INTRODUCTION

Metabolic syndrome (MetS) is associated with the development of non-communicable diseases such as cardiovascular disease, type 2 diabetes mellitus, and stroke [1,2]. MetS affects 20-30% of the adult population in Europe [3], and its prevalence in adults in the United States approximately 36.4% [4]. Similarly, studies have shown an increasing prevalence of MetS in the Korean population (from 24.9% in 1998 to 28.9% in 2013) [5]. These data suggest that MetS is increasingly becoming a public health issue that also leads to increased medical costs and other socioeconomic burdens [6].

Numerous studies have identified the risk factors for MetS in various domains, including investigations on lifestyle factors, physical activities, and dietary intake [7,8]. Studies have reported that the consumption of fruits and vegetables could reduce the risk of MetS [9,10]; a vegetarian diet was reported to be associated with a lower incidence of MetS in a previous study [11]. A meta-analysis also revealed that fruit and vegetable consumption was inversely associated with the risk of developing MetS [12].

In Korea, daily fruit and vegetable consumption decreased from 35.7% in 2007 to 26.2% in 2018 according to the 2018 National Health Statistics [13]; reduced intake of fruits and vegetables is, in turn, associated with a reduction in the intake of vitamins and minerals [14]. Interestingly, there is instead an increasing trend in the consumption of dietary supplements such as multivitamins [15].

According to studies based on animal models, clinical trials, and the biochemical analysis of new multivitamins, the intake of multivitamins with suitable proportions of vitamins B3 (niacin), B1 (thiamine), B2 (riboflavin), B6 (pyridoxine), and others with antioxidant activity can reduce symptom severity or the risk of chronic diseases, such as MetS [16]. It has also been reported that dietary supplementation with vitamin A (retinol and retinoic acid), vitamin E, and total antioxidant capacity vitamins decreased the prevalence of MetS and its component factors [17]. In this regard, there are insufficient findings in the literature to confirm whether the consumption of fruits and vegetables with concomitant use of multivitamins reduces the risk of MetS.

Hence, in this study, we aimed to examine whether concomitant multivitamin use and the adequate consumption of fresh vegetables and fruits are associated with MetS and its five components among Korean adults aged 40 years to 69 years through a study design based on the Health Examinees (HEXA) study.

## MATERIALS AND METHODS

### Study population

This investigation was based on the HEXA study, a large-scale community-based genomic cohort study in Korea that was implemented from 2004 to 2013. The specific details of the HEXA study design have been thoroughly described elsewhere [18]. In total, 173,357 individuals aged 40 years to 69 years were initially included in this survey. Among this population, we excluded individuals who had missing values for the measures of certain components of MetS (n= 6,022), individuals who reported doubtful energy intake values (< 800 or ≥ 4,000 kcal/day in male, < 500 or ≥ 3,500 kcal/day in female; n= 3,132) [19], those who did not report the use of a multivitamin supplement (n= 69,455), and individuals who had missing values needed for the adjusting of possible confounding variables such as body mass index (BMI), household income, education level, smoking status, drinking status, and physical activity (n= 5,200). Ultimately, 89,548 participants (28,828 male and 60,720 female) were included in this study.

### Definition of metabolic syndrome

MetS was defined in accordance with the National Cholesterol Education Program, Adult Treatment Panel III [20], and waist circumference (WC) criteria were based on the guidelines of the Korean Obesity Society [21]. Participants were diagnosed with MetS if they had 3 or more of the following 5 components: (1) increased WC, ≥ 90 cm in male and ≥ 85 cm in female; (2) elevated triglyceride (TG) levels, ≥ 150 mg/dL; (3) reduced levels of high-density lipoprotein cholesterol (HDL cholesterol), < 40 mg/dL in male and < 50 mg/dL in female; (4) elevated fasting blood glucose (FBG) levels, ≥ 100 mg/dL or using diabetes medication; (5) elevated blood pressure, systolic blood pressure (SBP) ≥ 130 mmHg or diastolic blood pressure (DBP) ≥ 85 mmHg or using hypertension medication.

### Dietary assessment and multivitamin use

Regular fruit and vegetable consumption was assessed using a self-administered 106-item food frequency questionnaire [22]. Participants reported the frequency and average amount of food or beverage items consumed during the previous year based on 3 standard serving sizes (0.5, 1.0, and 1.5 serving sizes). The consumption frequencies were classified into 14 categories, ranging from “never or seldom” to “3 times/day.”

In the current study, we included 12 fruit items as fresh fruits: strawberries, muskmelons/melons, watermelons, peaches/plums, bananas, persimmons, tangerines, pears, apples, oranges, grapes, and tomatoes. We classified “vegetable wrap/vegetable salad” as involving fresh vegetables intake. Daily vegetable and fruit intake was calculated by multiplying the frequency of consumption by the specific serving sizes. Each vegetable and fruit item was used to classify consumption into 3 main groups: fresh vegetables+ fruits, fresh vegetables, and fruits.

The total daily intake of energy and macronutrients (carbohydrates, proteins, and fats) were calculated using the food composition table of the Korean Health and Industry of Development Institute [23,24]. The amount or frequency of multivitamin use among the participants of this survey from 2004 to 2008 was classified into the following 6 categories: none, 1-3 pills or times/wk, 4-6 pills or times/wk, 1 pill or times/day, 2 pills or times/day, and ≥ 3 pills or times/day. For those who participated in this survey from 2009 to 2013, the amount or frequency of multivitamin use was calculated by multiplying the frequency of consumption by the amount per consumption. According to the results of the calculations (0 or not), participants were dichotomized as multivitamin users or non-users.

### Statistical analysis

All statistical analyses were performed using SAS version 9.4 (SAS Institute Inc., Cary, NC, USA), and a p-value < 0.05 was considered to indicate statistically significant. We performed analyses to estimate the odds ratios (ORs) and 95% confidence interval (CIs) of MetS and its components based on the quartiles (Q4) of fresh vegetable and fruit consumption by sex. Multivariable logistic regression analysis was performed after adjusting for potential confounders, such as age (continuous), BMI (except WC), household income (low, medium–low, medium–high, or high), education level (≤ elementary school, middle school, high school, or ≥ college), smoking status (never, past, or current), drinking status (never, past, or current), physical activity (yes or no), and energy intake (kcal/day, analyzed as a continuous variable). Linear trends across the quartiles were tested by assigning each participant the median value of the category and modeling the corresponding values as a continuous variable. Differences between categories were calculated using the chi-square test for categorical variables and generalized linear models for continuous variables. Stratified analyses were also performed to test whether observed associations relied on the use of multivitamins. In the stratified analyses, multivariable logistics models (adjusted for continuous and categorical confounders) were applied to assess the association between each 1-standard deviation (SD) increment of fruit and vegetable intake and MetS for multivitamin use. The p-value for interaction was tested by performing the likelihood ratio test. All these analyses were conducted separately by sex.

### Ethics statement

All participants voluntarily signed an informed written consent form prior to enrollment. The current study was performed in accordance with the guidelines specified in the Declaration of Helsinki, and the study protocol was approved by the local Institutional Review Board of the Ethics Committee of the Korean Genome and Epidemiology Study of the Korea National Institute of Health (IRB No. E-1503-103-657).

## RESULTS

The general characteristics of the study population by sex are presented in Table 1 in accordance with the quartiles of fresh vegetable and fruit consumption. Participants with higher fresh vegetable and fruit consumption also exhibited higher multivitamin intake, higher household income levels and education levels, and physical activity, and were less likely to be smokers or drinkers than those with the lowest fresh vegetable and fruit consumption (all p<0.001). Compared with male in the lowest quartile, those who were in the highest quartile of fresh vegetable and fruit intake were slightly older (p<0.001), whereas female in the highest quartile were slightly younger and had a higher BMI (p<0.001 and p=0.028, respectively). Analysis of daily energy and energy from macronutrients across the quartiles of the fresh vegetable and fruit consumption revealed that both male and female with higher fresh vegetable and fruit consumption had higher intakes of energy and energy proportions from proteins and fats than those with lower fresh vegetable and fruit consumption; however, energy intake from carbohydrates was inversely associated (all p<0.001). All selected covariates exhibited statistically significant differences among the fresh vegetable and fruit consumption categories (Table 1).

The biomarkers and prevalence of MetS and its components according to the quartiles of fresh vegetable and fruit intake are presented in Table 2. Male with higher fresh vegetable and fruit consumption had the lower HDL cholesterol levels (p<0.001), as well as the lower SBP and DBP (p=0.004 and < 0.001, respectively); however, WC (p=0.567), TG (p=0.147) and FBG levels (p=0.713) were not significantly associated with the consumption of fresh vegetables and fruits. Except for HDL cholesterol levels, SBP and DBP, female with higher consumption of fresh vegetables and fruits had the lower mean WC, TG levels, and FBG levels (p values are 0.001, 0.017, and < 0.001, respectively).

In the analysis of the prevalence of the 5 indicators of MetS in female, those who consumed more fresh vegetables and fruit had a lower prevalence of MetS and its five components (all p<0.001). However, in male, those who consumed more fresh vegetables and fruits only showed a lower prevalence of elevated TG levels (p=0.005), but a higher prevalence of reduced HDL cholesterol levels (p=0.020). Moreover, the prevalence of MetS (p=0.349), increased WC (p=0.970), or elevated FBG levels (p=0.717) did not show significant associations with fresh vegetable and fruit consumption (Table 2).

The associations between fresh vegetable, fruit, or fresh vegetable+fruit consumption and the prevalence of MetS and its components are presented in Table 3. Female in the highest quartile of fresh vegetable, fruit, and fresh vegetable+fruit consumption had lower ORs of MetS (OR, 0.79; 95% confidence interval [CI], 0.74 to 0.85; p<0.001 in the fresh vegetable group, OR, 0.93; 95% CI, 0.87 to 0.99; p<0.001 in the fruit group, and OR, 0.91; 95% CI, 0.85 to 0.98; p<0.001 in the fresh vegetable and fruit group) than those in the lowest quartile. Higher fresh vegetable consumption was also associated with a lower OR of individual MetS components, including increased WC (OR, 0.83; 95% CI, 0.77 to 0.89; p<0.001), elevated TG levels (OR, 0.86; 95% CI, 0.80 to 0.92; p<0.001), and reduced HDL cholesterol levels (OR, 0.83; 95% CI, 0.78 to 0.87; p<0.001). Similarly, higher fruit and fresh vegetable+fruit consumption was associated with lower ORs of increased WC (OR, 0.88; 95% CI, 0.83 to 0.95; p<0.001 in the fruit group and OR, 0.88; 95% CI, 0.82 to 0.94; p<0.001 in the fresh vegetable+fruit group). Among male, a significant association was observed between higher fresh vegetable consumption and the prevalence of MetS (OR, 0.88; 95% CI, 0.81 to 0.96; p<0.001). However, there was no significant association between other consumption categories and the prevalence of MetS, and regarding the components of MetS, only elevated blood pressure exhibited lower prevalence than the group with the lowest consumption (OR, 0.90; 95% CI, 0.84 to 0.97; p<0.001 in the fruit group and OR, 0.90; 95% CI, 0.84 to 0.97; p<0.001 in the fresh vegetable+fruit group) (Table 3).

**Figure 1.**
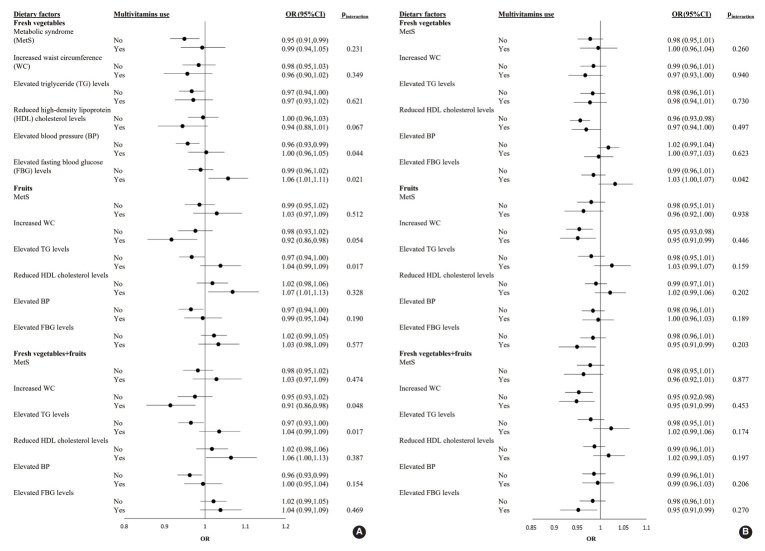
Association between vegetable and fruit consumption and MetS among male (A) and female (B) in the Health Examinees study stratified by multivitamin use. Vegetable and fruit consumption was modeled per standard deviation increment in intake. The models were adjusted by age, household income, drinking status, smoking status, educational level, physical activity level, income level, body mass index, and daily total energy intake. Multiplicative interactions on an OR scale were considered statistically significant if p<0.05. OR, odds ratio; CI, confidence interval.

The ORs and 95% CI of MetS for each 1-SD increment of fresh vegetable, fresh fruit, or fresh vegetable+fresh fruit consumption in male and female who were or were not taking multivitamins are shown in Figure 1. Among male participants, the adjusted OR for elevated FBG levels with a 1-SD increment in fresh vegetable intake was significantly higher in multivitamin users (OR, 1.06; 95% CI, 1.01 to 1.11) than in non-users (p_interaction_=0.021). Regarding the effects of fresh vegetables+fruits on increased WC, the adjusted OR was significantly lower in multivitamin users as consumption increased (OR, 0.91; 95% CI, 0.85 to 0.98; p_interaction_=0.048). Among female participants, there was no significant interaction for the prevalence of MetS and its 5 components between the 3 categories and multivitamin intake (all p_interaction_>0.05) (Figure 1).

## DISCUSSION

In the current cross-sectional study, we discovered that fruit and fresh vegetable consumption had an association with a lower prevalence of MetS. Considering that kimchi (a traditional fermented vegetable in Korea) is very frequently consumed in Korea, we additionally adjusted the model for kimchi intake, and the cross-sectional association remained (Supplementary Material 1). However, concomitant multivitamin use did not alter the relationship in either male or female with adequate fruit and vegetable consumption.

Our findings are similar to those of previous cross-sectional studies conducted in Korea that identified an inverse association between fruit consumption and the prevalence of MetS because of fruits’ phytochemicals and antioxidative vitamins [10,25]. Furthermore, several prospective epidemiological studies also demonstrated that a lower incidence of MetS was associated with higher fruit consumption due to the beneficial health-related components of fruits, such as antioxidants, phytochemicals, fiber, and minerals [26,27]. The slight difference in the association between vegetables and MetS compared with that in a previous Korean study may have resulted from the differences in vegetable inclusion criteria. In the current study, we included only fresh leafy vegetables and did not consider kimchi or tuber vegetables [10,26]. In a systematic review and meta-analysis, Shin et al. [28] reported that there were no significant associations between MetS and its 5 components and fruit and vegetable intake except for DBP. The result was inconsistent with ours, which may have been due to differences in subjects and exposures [28-32].

Fruits and vegetables are essential, and they are rich in fiber, minerals, vitamins, and certain phytochemicals [33]. The risk of MetS may be amplified by antioxidant deficiency accompanied by oxidative stress, which may lead to oxidative changes in the extracellular space, promoting endothelial dysfunction and cardiovascular damage [34]. A higher intake of antioxidants could reduce the oxidative stress associated with MetS [35,36]. In addition, as phytochemicals can increase the body’s production of insulin [37], the components of fruits and vegetables may play a role in preventing insulin resistance associated with MetS [35].

Although there are some multivitamin intake guidelines for certain populations, such as pregnant female, more recommendations or studies are needed for the general population [38]. The health benefits of multivitamin intake have been controversial. According to the National Institutes of Health State-of-the-Science Conference Statement, there is insufficient evidence to recommend multivitamins for the treatment or prevention of chronic diseases [39]. The Academy of Nutrition and Dietetics also noted in a 2009 position statement that there was no evidence that multivitamins are effective in preventing chronic disease and that it was preferable to maintain one’s health and to prevent chronic diseases by eating a variety of foods [40,41]. These statements are consistent with our findings that revealed no additional effects of multivitamin use in reducing the prevalence of MetS.

In the current sex-stratified, cross-sectional analysis, overall, sex differences did not play a major role in the effects of the three categories on the prevalence of MetS, except that certain variables were more likely to show statistical significance among female than among male. According to a previous study, the slight difference may result from the differences in musculoskeletal and adipose tissue and sex hormone levels [42-44].

The current study had some limitations. First, despite the finding of an inverse association between fresh vegetable and fruit intake and MetS, the limited cross-sectional data could not reveal causal associations. Second, we obtained the information regarding the quantity of fruits and vegetables consumed and multivitamin intake using a self-reported food frequency questionnaire, and there was no detailed information about whether the fruits and vegetables were processed; thus, dietary measurement errors were inevitable [45]. Finally, our study only included Korean adults aged 40-69 years; therefore, a complete analysis of Korean diets and their impact on health outcomes, such as MetS, would require a further evaluation with the inclusion of all age groups.

Higher intake of fresh vegetables and fruits was associated with a lower overall prevalence of MetS. However, there was no significant synergistic effect of concomitant multivitamin use in those with adequate consumption of fresh vegetables and fruits. In the future, we will focus on the longitudinal association between fresh vegetable and fruit intake and the incidence of MetS to investigate the impact of improved eating habits, with an emphasis on the increased consumption of a variety of fresh vegetables and fruits, on the prevention of MetS rather than promoting the use of multivitamins.

## Figures and Tables

**Table 1.** General characteristics of the study population according to quartiles (Q) of fresh vegetable and fruit consumption

Characteristics			Q1	Q2	Q3	Q4	p-value
Male (n=28,828), n			7,207	7,207	7,207	7,207	
	Age (yr)		54.31±9.07	54.25±8.93	54.36±8.98	54.7±8.87	<0.001
	Multivitamin intake (yes)		1,745 (24.2)	2,095 (29.1)	2,275 (31.6)	2,612 (36.2)	<0.001
	Body mass index (kg/m^2^)		24.30±2.77	24.38±2.74	24.33±2.72	24.46±2.66	0.154
	Household income level (high)		1,219 (22.1)	1, 452 (25.3)	1,597 (27.3)	1,918 (31.7)	<0.001
	Education level (≥college)		1,978 (27.4)	2,479 (34.4)	2,682 (37.2)	2,998 (41.6)	<0.001
	Smoking status (current)		2,590 (35.9)	2,282 (31.7)	2,125 (29.5)	1,799 (25.0)	<0.001
	Drinking status (current)		5,353 (74.3)	5,259 (73.0)	5,085 (70.6)	4,879 (67.7)	<0.001
	Physical activity (yes)		1,026 (14.2)	1,263 (17.5)	1,419 (19.7)	1,712 (23.7)	<0.001
	Nutrient intake						
		Energy (kcal)	1,631.33±420.77	1,779.49±440.47	1,874.02±447.41	2,075.32±493.67	<0.001
		Carbohydrates (% energy)	74.13±7.40	72.08±7.05	71.71±6.87	71.37±6.75	<0.001
		Proteins (% energy)	12.83±2.40	13.55±2.36	13.71±2.36	13.87±2.44	<0.001
		Fats (% energy)	13.05±5.45	14.37±5.16	14.58±4.98	14.76±4.81	<0.001
Female (n=60,720), n			15,180	15,180	15,180	15,180	
	Age (yr)		53.66±8.69	52.64±8.27	52.45±8.00	52.06±7.70	<0.001
	Multivitamin intake (yes)		4,234 (27.9)	5,189 (34.2)	5,547 (36.5)	6,270 (41.3)	<0.001
	Body mass index (kg/m^2^)		23.90±3.07	23.74±2.92	23.72±2.90	23.51±2.81	0.028
	Household income level (high)		1,893 (17.6)	2,544 (21.9)	2,986 (25.3)	3,464 (28.5)	<0.001
	Education level (≥college)		1,846 (12.2)	2,467 (16.2)	2,859 (18.8)	3,443 (22.7)	<0.001
	Smoking status (current)		474 (3.1)	370 (2.4)	261 (1.72%)	207 (1.4)	<0.001
	Drinking status (current)		4,685 (30.9)	4,804 (31.6)	4,572 (30.1)	4,463 (29.4)	<0.001
	Physical activity (yes)		2,131 (14.0)	2,480 (16.3)	2,786 (18.3)	3,286 (21.6)	<0.001
	Nutrient intake						
		Energy (kcal)	1,460.36±437.51	1,613.10±449.37	1,734.83±464.54	1,965.41±512.63	<0.001
		Carbohydrates (% energy)	74.66±7.68	72.70±7.39	72.18±7.25	71.99±7.03	<0.001
		Proteins (% energy)	13.01±2.59	13.65±2.55	13.85±2.55	13.83±2.57	<0.001
		Fats (% energy)	12.32±5.58	13.65±5.35	13.97±5.23	14.18±4.99	<0.001

Values are presented as adjusted mean±standard deviation or number (%).

**Table 2.** Biomarkers of MetS and its components according to quartiles (Q) of fresh vegetable and fruit consumption

Variables^1^			Q1	Q2	Q3	Q4	p-value
Male (n=28,828)			7,207	7,207	7,207	7,207	
	WC (cm)		86.03±7.60	86.17±7.45	86.06±7.48	86.18±7.38	0.567
	TG level (mg/dL)		155.20±120.50	152.61±113.93	149.43±104.65	145.69±100.89	0.143
	HDL cholesterol level (mg/dL)		50.63±13.59	49.91±11.91	49.57±11.90	49.56±11.59	<0.001
	FBG level (mg/dL)		99.35±26.53	99.41±23.89	98.87±23.50	99.03±23.37	0.713
	SBP (mmHg)		126.88±15.50	126.30±15.34	125.89±14.90	125.55±14.66	0.004
	DBP (mmHg)		79.62±10.17	78.95±10.08	78.66±9.83	78.83±9.81	<0.001
	Prevalence						
		MetS	1,863 (25.8)	1,813 (25.2)	1,788 (24.8)	1,776 (24.6)	0.349
		Increased WC	2,251 (31.2)	2,265 (31.4)	2,260 (31.4)	2,278 (31.6)	0.970
		Elevated TG levels	2,753 (38.2)	2,687 (37.3)	2,664 (37.0)	2,550 (35.4)	0.005
		Reduced HDL cholesterol levels	1,237 (17.2)	1,315 (18.2)	1,377 (19.1)	1,341 (18.6)	0.020
		Elevated FBG levels	2,473 (34.3)	2,490 (34.5)	2,427 (33.7)	2,473 (34.3)	0.717
		Elevated BP	3,427 (47.5)	3,316 (46.0)	3,242 (45.0)	3,182 (44.1)	<0.001
Female (n=60,720)			15,180	15,180	15,180	15,180	
	WC (cm)		79.70±8.47	79.06±8.15	78.83±8.10	78.15±7.92	0.001
	TG level (mg/dL)		118.66±79.80	114.12±74.04	112.09±72.83	111.36±73.06	0.017
	HDL cholesterol level (mg/dL)		55.92±12.90	56.14±12.69	56.47±12.72	56.80±12.83	0.471
	FBG level (mg/dL)		94.27±22.09	93.12±21.55	92.76±18.27	92.17±18.10	<0.001
	SBP (mmHg)		121.99±16.70	121.31±16.12	120.88±15.80	119.90±15.44	0.160
	DBP (mmHg)		75.59±10.24	75.13±10.07	75.05±9.98	74.77±9.78	0.616
	Prevalence						
		MetS	3,801 (25.0)	3,449 (22.7)	3,258 (21.5)	2,890 (19.0)	<0.001
		Increased WC	7,350 (48.4)	6,894 (45.4)	6,663 (43.9)	6,171 (40.6)	<0.001
		Elevated TG levels	3,481 (22.9)	3,194 (21.0)	3,038 (20.0)	2,966 (19.5)	<0.001
		Reduced HDL cholesterol levels	5,089 (33.5)	4,943 (32.6)	4,795 (31.6)	4,640 (30.6)	<0.001
		Elevated FBG levels	3,365 (22.2)	3,090 (20.4)	3,054 (20.1)	2,752 (18.1)	<0.001
		Elevated BP	5,155 (34.0)	4,925 (32.4)	4,756 (31.3)	4,430 (29.2)	<0.001

Values are presented as adjusted mean±standard deviation or number (%). MetS, metabolic syndrome; WC, waist circumference; TG, triglyceride; HDL, high-density lipoprotein; SBP, systolic blood pressure; DBP, diastolic blood pressure; FBG, fasting blood glucose; BP, blood pressure.

1 Increased WC: WC ≥90 cm in male, ≥85 cm in female; Elevated TG level: ≥150 mg/dL; Reduced HDL cholesterol level: <40 mg/dL in male, <50 mg/dL in female; Elevated BP: SBP ≥130 mmHg or DBP ≥85 mmHg or taking hypertensive medication; Elevated FBG level: fasting glucose ≥100 mg/dL or taking diabetes medication.

**Table 3.** Associations between vegetable/fruit consumption1 and MetS among Health Examinees study participants

Variables^1,2^			Q1	Q2	Q3	Q4	p for trend
Male (n=28,828)							
	Fresh vegetables (n)		7,012	7,039	6,661	8,116	
		Range (Min-Max), g/day	0.00–0.00	0.83–2.50	4.17–6.25	10.71–225.00	
		MetS	1.00 (reference)	1.03 (0.95, 1.12)	0.91 (0.83, 0.99)	0.88 (0.81, 0.96)	<0.001
		Increased WC	1.00 (reference)	0.95 (0.86, 1.05)	0.86 (0.78, 0.95)	0.86 (0.78, 0.95)	0.003
		Elevated TG levels	1.00 (reference)	0.97 (0.90, 1.04)	1.00 (0.93, 1.08)	0.97 (0.90, 1.05)	0.002
		Reduced HDL cholesterol levels	1.00 (reference)	1.02 (0.93, 1.11)	0.99 (0.91, 1.09)	1.04 (0.95, 1.13)	0.435
		Elevated BP	1.00 (reference)	0.95 (0.88, 1.01)	0.94 (0.87, 1.01)	0.87 (0.82, 0.94)	<0.001
		Elevated FBG levels	1.00 (reference)	1.04 (0.97, 1.12)	1.02 (0.94, 1.09)	1.01 (0.94, 1.08)	0.742
	Fruits (n)		7,207	7,207	7,207	7,207	
		Range (Min-Max), g/day	0.00–52.50	52.50–114.90	114.91–223.54	223.60–3,410.00	
		MetS	1.00 (reference)	0.96 (0.89, 1.05)	1.01 (0.92, 1.09)	0.99 (0.90, 1.08)	<0.001
		Increased WC	1.00 (reference)	1.00 (0.91, 1.10)	1.01 (0.91, 1.11)	0.95 (0.85, 1.05)	0.002
		Elevated TG levels	1.00 (reference)	0.97 (0.90, 1.04)	1.00 (0.93, 1.08)	0.97 (0.90, 1.05)	0.002
		Reduced HDL cholesterol levels	1.00 (reference)	1.07 (0.98, 1.17)	1.21 (1.11, 1.32)	1.15 (1.05, 1.26)	0.442
		Elevated BP	1.00 (reference)	0.95 (0.88, 1.01)	0.94 (0.88, 1.01)	0.90 (0.84, 0.97)	<0.001
		Elevated FBG levels	1.00 (reference)	1.00 (0.93, 1.07)	0.99 (0.93, 1.07)	1.018 (0.94, 1.10)	0.767
	Fresh vegetables and fruits (n)		7,207	7,207	7,207	7,207	
		Range (Min-Max), g/day	0.00–57.75	57.77–122.28	122.28–233.13	233.15–3,460.00	
		MetS	1.00 (reference)	0.97 (0.90, 1.06)	1.00 (0.92, 1.09)	0.98 (0.90, 1.07)	<0.001
		Increased WC	1.00 (reference)	0.98 (0.89, 1.08)	1.03 (0.93, 1.13)	0.93 (0.84, 1.03)	0.002
		Elevated TG levels	1.00 (reference)	0.99 (0.92, 1.06)	1.01 (0.94, 1.09)	0.97 (0.90, 1.05)	0.002
		Reduced HDL cholesterol levels	1.00 (reference)	1.10 (1.01, 1.20)	1.18 (1.08, 1.29)	1.16 (1.05, 1.27)	0.443
		Elevated BP	1.00 (reference)	0.95 (0.89, 1.02)	0.93 (0.87, 1.00)	0.90 (0.84, 0.97)	<0.001
		Elevated FBG levels	1.00 (reference)	1.02 (0.95, 1.09)	0.99 (0.92, 1.07)	1.02 (0.94, 1.10)	0.768
Female (n=60,720)							
	Fresh vegetables (n)		15,317	18,857	15,159	11,387	
		Range (Min-Max), g/day	0.00–0.83	1.67–4.17	5.36–10.71	12.50–225.00	
		MetS	1.00 (reference)	0.89 (0.84, 0.94)	0.89 (0.84, 0.95)	0.79 (0.74, 0.85)	<0.001
		Increased WC	1.00 (reference)	0.88 (0.83, 0.94)	0.89 (0.84, 0.95)	0.83 (0.77, 0.89)	<0.001
		Elevated TG levels	1.00 (reference)	0.93 (0.88, 0.98)	0.93 (0.87, 0.98)	0.86 (0.80, 0.92)	<0.001
		Reduced HDL cholesterol levels	1.00 (reference)	0.94 (0.90, 0.98)	0.92 (0.87, 0.96)	0.83 (0.78, 0.87)	<0.001
		Elevated BP	1.00 (reference)	1.02 (0.97, 1.07)	1.00 (0.95, 1.05)	1.01 (0.95, 1.07)	0.927
		Elevated FBG levels	1.00 (reference)	0.93 (0.88, 0.98)	0.93 (0.87, 0.98)	0.94 (0.88, 1.00)	0.241
	Fruits (n)		15,180	15,180	15,180	15,180	
		Range (Min-Max), g/day	0.00–83.65	83.66–173.63	173.63–323.93	323.93–4,480.00	
		MetS	1.00 (reference)	1.02 (0.96, 1.08)	0.97 (0.91, 1.03)	0.93 (0.87, 0.99)	<0.001
		Increased WC	1.00 (reference)	0.97 (0.91, 1.03)	0.92 (0.86, 0.98)	0.88 (0.83, 0.95)	<0.001
		Elevated TG levels	1.00 (reference)	0.97 (0.92, 1.03)	0.94 (0.90, 1.00)	0.98 (0.92, 1.04)	<0.001
		Reduced HDL cholesterol levels	1.00 (reference)	1.04 (0.99, 1.10)	1.00 (0.95, 1.05)	1.03 (0.97, 1.08)	<0.001
		Elevated BP	1.00 (reference)	1.03 (0.98, 1.09)	1.00 (0.95, 1.06)	0.97 (0.91, 1.02)	0.994
		Elevated FBG levels	1.00 (reference)	0.97 (0.92, 1.03)	0.98 (0.92, 1.04)	0.92 (0.87, 0.98)	0.144
	Fresh vegetables and fruits (n)		15,180	15,180	15,180	15,180	
		Range (Min-Max), g/day	0.00–90.27	90.27–182.95	182.95–334.91	334.91–4,481.67	
		MetS	1.00 (reference)	1.00 (0.94, 1.06)	0.95 (0.90, 1.01)	0.91 (0.85, 0.98)	<0.001
		Increased WC	1.00 (reference)	0.96 (0.90, 1.02)	0.91 (0.85, 0.97)	0.88 (0.82, 0.94)	<0.001
		Elevated TG levels	1.00 (reference)	0.97 (0.92, 1.03)	0.94 (0.88, 0.99)	0.98 (0.92, 1.04)	<0.001
		Reduced HDL cholesterol levels	1.00 (reference)	1.02 (0.97, 1.07)	1.00 (0.95, 1.05)	1.01 (0.95, 1.06)	<0.001
		Elevated BP	1.00 (reference)	1.03 (0.98, 1.09)	1.00 (0.95, 1.06)	0.97 (0.91, 1.02)	0.996
		Elevated FBG levels	1.00 (reference)	0.97 (0.92, 1.03)	0.99 (0.93, 1.05)	0.93 (0.87, 0.99)	0.143

Values are presented as adjusted odds ratio (95% confidence interval). Model was adjusted for age, body mass index, educational level, physical activity, smoking status, drinking status and total energy intake. MetS, metabolic syndrome; Min, minimum; Max, maximum; WC, waist circumference; TG, triglyceride; HDL, high-density lipoprotein; SBP, systolic blood pressure; DBP, diastolic blood pressure; FBG, fasting blood glucose; BP, blood pressure.

1 Fresh vegetables: only fresh vegetable; Fruits: only fruit; Fresh vegetables and fruits: both fresh vegetables+fruits.

2 Increased WC: WC ≥90 cm in male, ≥85 cm in female; Elevated TG level: ≥150 mg/dL; Reduced HDL cholesterol level, <40 mg/dL in male, <50 mg/dL in female; Elevated BP: SBP ≥130 mmHg or DBP ≥85 mmHg or taking hypertensive medication; Elevated FBG level: fasting glucose ≥100 mg/dL or taking diabetes medication.

## SUPPLEMENTARY MATERIALS

Supplementary Material 1. Adjusted ORs for MetS and its components according to fresh vegetable intake in model 2
