# Supplementary material for: The association between fruit and vegetable consumption and metabolic syndrome in Korean adults: does multivitamin use matter?
Source: Epidemiol Health. 2022 Apr 19;44:e2022039. doi: 10.4178/epih.e2022039 (PMC9684005; doi:10.4178/epih.e2022039)
Supplement: Supplementary Material 1. — Adjusted ORs for MetS and its components according to fresh vegetable intake in model 2 [file epih-44-e2022039-suppl.docx]

The association between fruit and vegetable consumption and metabolic syndrome in Korean adults: does multivitamin use matter?

Supplementary Material 1. Adjusted ORs for MetS and its components according to fresh vegetable intake in model 2

|  | Q1 | Q2 | Q3 | Q4 | *P* for trend |
| --- | --- | --- | --- | --- | --- |
| Men (n= 28 828) |  |  |  |  |  |
| Fresh vegetables | 7 012 | 7 039 | 6 661 | 8 116 |  |
| Range (min–max), g/day | 0.00–0.00 | 0.83–2.50 | 4.17–6.25 | 10.71–225.00 |  |
| MetS | 1.00 (ref.) | 1.034 (0.951,1.124) | **0.905 (0.830,0.988)** | **0.875 (0.804,0.952)** | **0.0001** |
| Increased WC | 1.00 | 0.953 (0.866,1.050) | **0.858 (0.777,0.948)** | **0.858 (0.779,0.944)** | **0.0021** |
| Elevated TG levels | 1.00 | 0.956 (0.889,1.027) | **0.909 (0.844,0.978)** | **0.883 (0.822,0.949)** | **0.0010** |
| Reduced HDL cholesterol levels | 1.00 | 1.022 (0.936,1.117) | 0.994 (0.908,1.089) | 1.035 (0.948,1.131) | 0.4851 |
| Elevated BP | 1.00 | 0.949 (0.886,1.017) | 0.939 (0.875,1.008) | **0.869 (0.811,0.932)** | **<.0001** |
| Elevated FBG levels | 1.00 | 1.044 (0.971,1.121) | 1.017 (0.945,1.095) | 1.003 (0.933,1.078) | 0.6603 |
|  |  |  |  |  |  |
| Women (n= 60 720) |  |  |  |  |  |
| Fresh vegetables | 15 317 | 18 857 | 15 159 | 11 387 |  |
| Range (min–max), g/day | 0.00–0.83 | 1.67–4.17 | 5.36–10.71 | 12.50–225.00 |  |
| MetS | 1.00 | **0.885 (0.836,0.936)** | **0.886 (0.834,0.942)** | **0.786 (0.734,0.843)** | **<.0001** |
| Increased WC | 1.00 | **0.884 (0.832,0.938)** | **0.89 (0.835,0.949)** | **0.825 (0.769,0.886)** | **<.0001** |
| Elevated TG levels | 1.00 | **0.925 (0.877,0.976)** | **0.923 (0.871,0.978)** | **0.852 (0.798,0.910)** | **<.0001** |
| Reduced HDL cholesterol levels | 1.00 | **0.939 (0.896,0.984)** | **0.914 (0.869,0.961)** | **0.821 (0.776,0.869)** | **<.0001** |
| Elevated BP | 1.00 | 1.017 (0.969,1.068) | 0.997 (0.946,1.051) | 1.001 (0.944,1.060) | 0.7348 |
| Elevated FBG levels | 1.00 | **0.929 (0.880,0.980)** | **0.924 (0.871,0.980)** | **0.936 (0.877,0.999)** | 0.9980 |

Model 2 was adjusted by age, BMI, household income, educational level, physical activity, smoking status, drinking status, total energy intake, and kimchi intake.

BP, blood pressure; FBG, fasting blood glucose; HDL, high-density lipoprotein; MetS, metabolic syndrome; SBP, systolic blood pressure; TG, triglyceride; WC, waist circumference

*p* < 0.001
